# Whole-Genome Sequence of the *Mycoplasma* (*Mesomycoplasma*) *hyorhinis* DSM 25591 Type Strain

**DOI:** 10.1128/MRA.00164-21

**Published:** 2021-04-22

**Authors:** Lisa Käbisch, Anne-Kathrin Schink, Dennis Hanke, Torsten Semmler, Corinna Kehrenberg, Stefan Schwarz

**Affiliations:** a Institute of Microbiology and Epizootics, Centre for Infection Medicine, Department of Veterinary Medicine, Freie Universität Berlin, Berlin, Germany; b Institute for Veterinary Food Science, Justus-Liebig-Universität Gießen, Gießen, Germany; c NG1-Microbial Genomics, Robert Koch Institute, Berlin, Germany; Indiana University, Bloomington

## Abstract

The whole-genome sequence of the type strain *Mycoplasma* (*Mesomycoplasma*) *hyorhinis* DSM 25591 is reported and compared to the available sequences of the corresponding type strains from other strain collections to ascertain conformity. Knowledge of the identity of type strains is of importance for their application in standardized test systems.

## Abstract (precis)

The whole-genome sequence of the type strain *Mycoplasma* (“*Mesomycoplasma*”) *hyorhinis* DSM 25591 is reported and compared to the available sequences of the corresponding type strains from other strain collections to ascertain conformity. Knowledge of the identity of type strains is of importance for their application in standardized test systems.

## ANNOUNCEMENT

Due to reclassification of the *Mollicutes*, the assignment of Mycoplasma hyorhinis to the novel genus *Mesomycoplasma* is under discussion (1–3). The American Type Culture Collection (ATCC), the National Collection of Type Cultures (NCTC), and the German Collection of Microorganisms and Cell Cultures GmbH (DSMZ) provide defined type strains. Investigating the degree of identity between type strains of different collections, we subjected *Mycoplasma* (*Mesomycoplasma*) *hyorhinis* DSM 25591 from the DSMZ to whole-genome sequencing. The obtained sequence was compared to the complete genome sequence of *M. hyorhinis* NCTC 10130 (GenBank accession number LS991950.1) and the partially available sequence of *M. hyorhinis* ATCC 17981 (NZ_KB911485.1). Prior to DNA isolation with the MasterPure complete DNA and RNA purification kit (Epicentre, Madison, WI, USA), *M. hyorhinis* DSM 25591 was cultured using modified Friis broth for 5 days at 37°C in a humidified, 7.5% CO_2_ atmosphere (4, 5). The DNA was quantified using a Qubit 3.0 fluorometer (double-stranded DNA broad-range assay kit; Thermo Fisher Scientific, Waltham, MA, USA), and its quality was assessed by spectral analysis (NanoDrop spectrophotometer; Thermo Fisher Scientific). Short-read sequencing was performed on an Illumina MiSeq sequencer with the MiSeq reagent kit v3 (Illumina, Inc., San Diego, CA, USA) and the Nextera XT library preparation kit (Illumina), resulting in 300-bp paired-end reads and a >90-fold coverage. In addition, long-read sequencing was performed using Oxford Nanopore MinION (Oxford Nanopore Technologies [ONT], Oxford, UK). MinION one-dimensional (1D) libraries were constructed using the SQK-RBK004 kit (ONT) and loaded according to the manufacturer’s instructions onto an R9.4 flow cell. Sequencing data were collected for 48 h. More detailed information on the sequencing parameters is provided in Table 1. A closed genome sequence was generated by a *de novo* hybrid assembly using a combination of short and long reads with Unicycler v0.4.7 (9). A draft genome assembly created with SPAdes v3.12 and consecutive connection of contigs, using the long reads from MinION, resulted in a single circular chromosomal DNA of 839,538 bp with a G+C content of 25.9% (10). Default parameters were used for the applied software.

**TABLE 1:** Sequencing characteristics of *M. hyorhinis* DSM 25591

Technology	Total no. of reads	Mean read length (bp)	Quality control	Adapter trimming	Error correction	Base calling	Avg genome coverage (×)	Amt of DNA used (ng)	SRA accession no.
Illumina MiSeq	1,500,074	285	FastQC (6)	Flexbar v3.0.3 (7)	BayesHammer (8)	NA	437	1	SRX9929725
Nanopore MinION	112,219	5,324	FastQC (6)	NA^*a*^	NA	Guppy	711	400	SRX9929726

a NA, not applicable.

**FIG 1:**
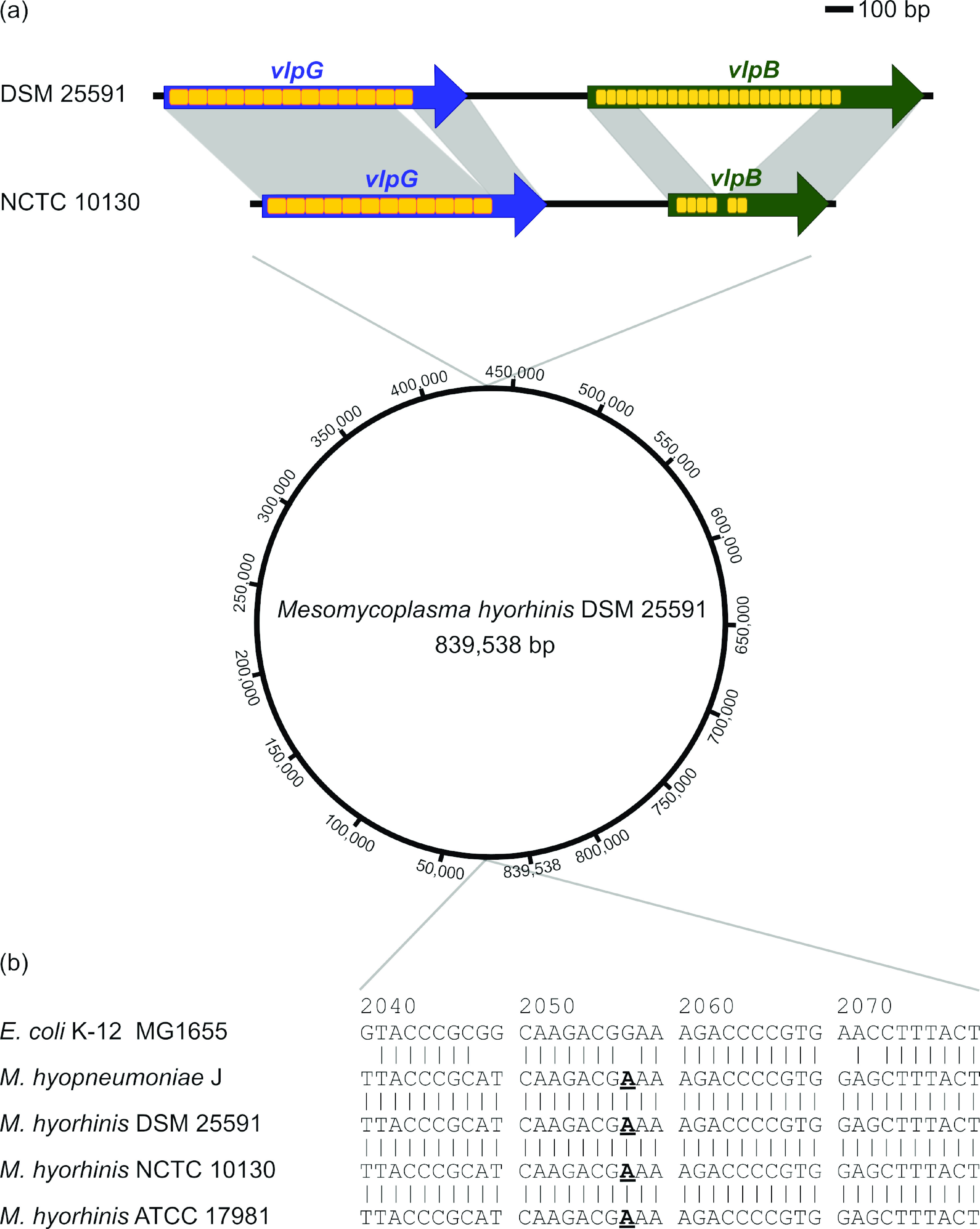
Presentation of the total genome sequence of *Mycoplasma* (*Mesomycoplasma*) *hyorhinis* DSM 25591 as a circular closed chromosomal DNA molecule of 839.538 bp, deposited in GenBank under the accession number CP064323. (a) Comparison of the *vlpG* and *vlpB* genes of *M. hyorhinis* DSM 25591 (CP064323) with those of *M. hyorhinis* NCTC 10130 (LS991950.1). The genes are shown as arrows, with the arrowheads indicating the direction of transcription. The individual repeats are displayed as boxes. Sequence similarities of the genes are indicated by gray shading. Compared to NCTC 10130, strain DSM 25591 showed 13 instead of 12 repeats of 66 bp (ORF position, 440934 to 442004) in *vlpG* and 24 repeats of 36 bp (ORF position, 442427 to 443614) instead of 6 complete and 1 partial repeat in v*lpB.* (b) Multisequence alignment of the 23S rRNA regions involved in macrolide resistance of M. hyopneumoniae J (NC_007295.1), *M. hyorhinis* DSM 25591 (CP064323), *M. hyorhinis* NCTC 10130 (LS991950.1), and *M. hyorhinis* ATCC 17981 (NZ_KB911485.1) compared to that of E. coli K-12 MG1655 (NC_000913.3). The nucleotide transition presumably conferring resistance to 14-membered macrolides is marked in bold and underlined.

Comparison with the sequence of *M. hyorhinis* NCTC 10130 showed an identity of 99.8%. The incomplete sequence of *M. hyorhinis* ATCC 17981 matched our sequence with an identity of 99.1 to 100%, depending on the respective contig. Two open reading frames (ORFs) within the regions encoding variable lipoproteins (Vlps) were further analyzed by PCR and Sanger sequencing (LGC Genomics, Berlin, Germany), as they differed from those in the *M. hyorhinis* NCTC 10130 sequence and were not included in the available sequence of *M. hyorhinis* ATCC 17981 (Fig. 1a). The number of repeats is known to potentially differ from generation to generation (11–13).

Analysis of the 23S rRNA of *M. hyorhinis* DSM 25591 identified a G2057A (Escherichia coli numbering) transition. In *Mycoplasma* (*Mesomycoplasma*) *hyopneumoniae*, transitions at nucleotide G2057 confer resistance to 14-membered macrolides (14). Therefore, the intrinsic resistance to erythromycin reported in the literature in *M. hyorhinis* might be explained by the G2057A transition, which is also seen in other available sequences deposited in the NCBI database (Fig. 1b) (14–17).

### Data availability.

The complete genome sequence of Mycoplasma hyorhinis DSM 25591 has been deposited in GenBank under the accession number CP064323. The version described in this paper is the first version, CP064323.1, annotated using the NCBI Prokaryotic Genome Annotation Pipeline (PGAP). The associated BioProject accession number is PRJNA674728; the SRA numbers are SRX9929726 and SRX9929725.
